# Microlearning through TikTok in Higher Education. An evaluation of uses and potentials

**DOI:** 10.1007/s10639-023-11904-4

**Published:** 2023-06-02

**Authors:** David Conde-Caballero, Carlos A. Castillo-Sarmiento, Inmaculada Ballesteros-Yánez, Borja Rivero-Jiménez, Lorenzo Mariano-Juárez

**Affiliations:** 1 Department of Nursing, Faculty of Nursing and Occupational Therapy, University of Extremadura, 10003 Cáceres, Spain; 2 Department of Nursing, Physiotherapy and Occupational Therapy, School of Physiotherapy and Nursing, University of Castilla-La Mancha, 45071 Toledo, Spain; 3 Regional Center for Biomedical Research, University of Castilla-La Mancha, 02008 Albacete, Spain; 4 Department of Inorganic and Organic Chemistry and Biochemistry, School of Medicine, University of Castilla-La Mancha, 13071 Ciudad Real, Spain; 5 Department of Business Management and Sociology, Teacher Training College, University of Extremadura, 10.003 Cáceres, Spain

**Keywords:** Digital learning technologies, e-learning, microlearning, social media, technology acceptance, TikTok

## Abstract

While social media is evolving rapidly, understanding its underlying and persistent features with the potential to support high-quality learning would provide opportunities to enhance competence acquisition and collaborative work in higher education. Moreover, the adoption of tools that students already use in their everyday lives facilitates the integration of new forms of learning. In this context, we have developed an initiative to disseminate content through TikTok in three modules of the Bachelor's Degree in Nursing course, with the aim of promoting quality learning through these microlearning environments. To this end, we have implemented these learning environments and evaluated the users’ perceptions, as well as their level of acceptance of the technology according to the Technology Acceptance Model. Overall, our results show high levels of satisfaction with regard to engagement and the content generated, as well as in terms of the acceptance of the technology. Our results do not show gender-specific variations, but we did detect slight variations depending on the subject in which the microlearning tool was deployed. Although for the most part these variations do not change the participants' assessment of their experience, it will be necessary in the future to determine the underlying reasons for these variations. In addition, our results suggest that it is possible to design a content creation system to promote quality learning through microlearning that can be transferred to other subjects, at least in the Bachelor's Degree in Nursing.

**Supplementary Information:**

The online version contains supplementary material available at 10.1007/s10639-023-11904-4.

## Introduction

In the last decade, the spread of social media and its use in pedagogical strategies has changed the paradigm of the teaching–learning process through the incorporation of internet-based resources. Indeed, more recently, the need for distance learning during the early months of the COVID-19 pandemic placed extra emphasis on this shift (Medina et al., 2019; Petronzi & Petronzi, 2020; Rose, 2020). As part of this “digital revolution” (Kaplan & Haenlein, 2016) a large number of teachers are striving to develop forms of active learning that promote critical thinking and a high degree of autonomous learning, particularly in the health sciences where most of the quality research has been focused, including through the introduction of more established technological tools such as blogs (Conde-Caballero et al., 2019) and wikis (Zitzelsberger et al., 2015) in higher education. Nevertheless, there are certain considerations which are specific to the integration of social media in higher education courses and the objectives of doing so. Several studies have delivered structured teaching through social media platforms (Guckian & Spencer, 2019; Hsieh et al., 2019; Webb et al., 2015), while others have focused more on promoting public engagement with the material covered by a course (López-Goñi & Sánchez-Angulo, 2018). Although there are undeniable barriers and limitations, various social media applications have emerged, supported by advanced web tools and the proliferation of smart mobile devices. In this context, the use of social media platforms such as Twitter (Chawinga, 2017; Roberts et al., 2016), Facebook (Pander et al., 2014) and Instagram (Reyna, 2021) in higher education seemed inevitable. Naturally, the development of new technological tools opens a range of new, unexplored pedagogical possibilities. However, the study of the application of these new tools in teaching has received less attention due to the availability of other options which, although already established, differ in nature.

Some studies (Pickering & Bickerdike, 2017; Webb et al., 2015) have shown a positive correlation between the degree of social media utilization and test score; others, however, suggest that time spent on social media has no impact (Manca & Ranieri, 2016). Although there is not yet any consensus on whether the use of social media correlates with academic results, the argument for the integration of social media into higher education practice is supported from multiple theoretical standpoints, particularly in health sciences education (Guckian et al., 2021; Whyte & Hennessy, 2017), including in the development of rapid, accessible virtual communities of practice (McLoughlin et al., 2018). It has, therefore, been suggested that the adoption of familiar tools already used by students can improve engagement, participation and motivation (Latif et al., 2019). Furthermore, students report changes in the student–educator relationship when social media is introduced to the classroom (Fischer et al., 2018; Raiman et al., 2017). Finally, social media initiatives make students feel more comfortable asking questions to their peers, compared to asking questions in a clinical setting (Ravindran et al., 2014). From the perspective of the educator, social media platforms have been employed to cover a wide range of pedagogical possibilities, including promoting small-group learning (Cole et al., 2017), empathy and self-efficacy (Anksorus & Bradley, 2020), medical professionalism (Hsieh et al., 2019), collaborative learning (Khan et al., 2021) and communication and learning (de Peralta et al., 2019).

One of the pedagogical applications of social media that has received the least attention is the promotion of microlearning, partly due to the lack of tools to properly support it (Sozmen, 2022). In the context of education in the health professions, microlearning (the acquisition of knowledge or skills in the form of small units) has been demonstrated to have a positive effect on students’ knowledge and confidence in performing procedures, knowledge retention, study habits, and engagement in collaborative learning (De Gagne et al., 2019). Although technology offers various media through which microlearning activities may be deployed, the use of short videos has already been recognised to enhance student learning in online education (Hsin & Cigas, 2013).

One potentially interesting tool is the video-sharing social media platform TikTok, which has experienced swift growth in use since its launch in 2016, and has enjoyed a rapid rise in popularity (Bhandari & Bimo, 2022). One of the reasons for its relatively quick success is its simple interface which allows users to easily create, edit and share audio-visual content (up to 3 min long) (Garg & Pahuja, 2020). The platform provides simple tools, along with image, audio and video databases, that allow users to generate videos of great complexity and originality. Although it was initially perceived by many as a platform that would be used almost exclusively by the younger generation, nowadays its users represent a broad cross-section of society, producing a wide variety of content (Bhandari & Bimo, 2022). Some experiences have been reported of the use of TikTok in teaching for pedagogical purposes (Radin & Light, 2022; Thornton, 2022), such as in dance (Cervi, 2021; Escamilla-Fajardo et al., 2021) and medical education (Comp et al., 2020). TikTok videos offer one means to help bridge the divide between professor and students and to provide students with a fun and relaxed way to keep up to date with all course related materials (Rand & Brushett, 2021). The platform is also a useful health promotion tool, as illustrated by its use in messaging to encourage the use of face masks during the Covid-19 pandemic (Basch et al., 2021).

In order to realise the full potential of these tools to promote learning through microlearning, we must be aware of the barriers and limitations in the pedagogical context in which this pedagogical innovation is to be implemented, while at the same time understanding the benefits that these tools can offer. On this basis, the present work aims to contribute to this objective by adding to the existing knowledge in the field of health-science teaching.

## Objectives

This project aims to critically analyse the wide range of pedagogical possibilities and limitations of the use of TikTok (one of the social networks with the greatest penetration in the university community), to promote TikTok-based microlearning environments, and thus to expand the body of evidence about the potential of TikTok in health sciences education.

Therefore, our main objective is to share our pedagogical experience, based on the design of a TikTok-based microcontent dissemination programme in several modules of the degree in nursing. It is hoped these experiences can be transferred to other teaching environments related to the health sciences.

A secondary objective of this work is to evaluate qualitatively and quantitatively the participants’ degree of satisfaction with several aspects of the teaching design used, as well as their acceptance of the technology. More specifically, our assessment of qualitative aspects covers more abstract constructs such as student engagement, the creation of learning communities and the promotion of creativity.

## Methodology

### Study design

**Fig. 1:**
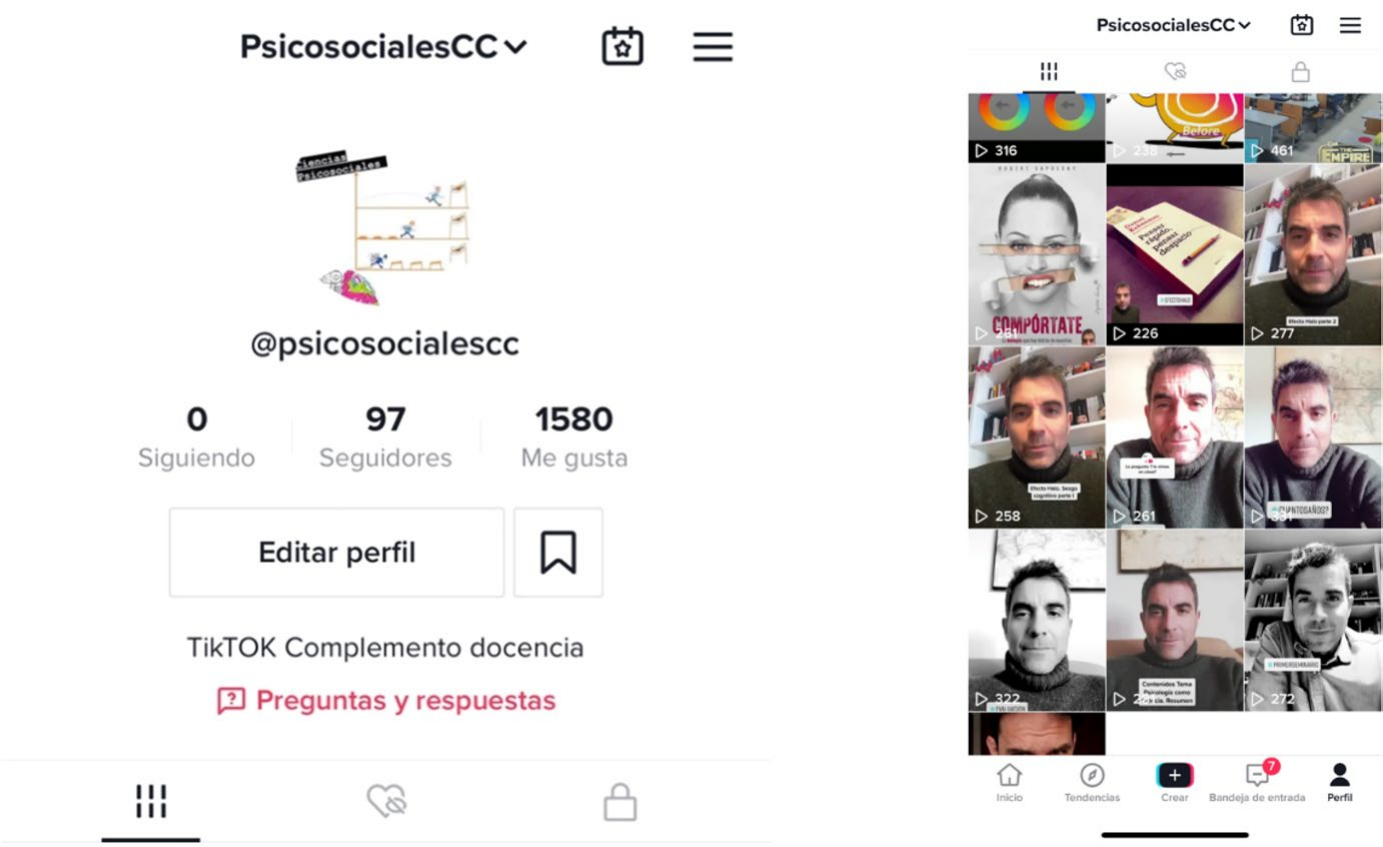
Screenshots of the Psychosocial Sciences Applied to Nursing subject TikTok account

The investigation was designed as a longitudinal quasi-experimental study and was conducted between February 7th and May 13th, 2022. The teacher responsible for the subject created a private TikTok account especially for the subject (i.e., they did not use their personal profile); Fig. 1 shows screenshots of one of these accounts as an example. Content was uploaded to this account throughout the study at a rate of at least 4 videos per week. The content uploaded to the TikTok account predominantly fell into one of two categories, which was indicated by a corresponding hashtag (#) in the description of the video:

- Content directly related to the course curriculum (#content), providing additional examples, historical curiosities, examples of assessments and reviews of topics.
- Complementary content related to cross-curricular issues that arose during the classes (#complementary), book recommendations, commentary on current news, etc.

Each of these categories is aligned with one of the outcomes that the use of the platform in this study was intended to support: 1) increasing participation and bridging the gap between teacher and student, 2) improving the acquisition of subject content and competences, 3) developing the processes of evaluation and self-evaluation through exercises presented in the videos, and 4) disseminating content which was related to the subject but which went beyond the syllabus, with the intention of encouraging interest and self-learning. The relationship between these objectives and the hashtags used are shown in Table 1 along with examples of videos created for this study.

**Table 1:** Relationship between intended outcomes and the hashtag used

Intended outcome	Example	Hashtag used in example
1) Increase participation	Video showing various food products and challenging students to guess what proportion of certain ingredients they contain	#complementary
2) Improve acquisition of competences	Video presenting the key points of the mandatory information that must appear on the labelling of food products	#content
3) Evaluation	Video in which the teacher poses a multiple-choice question with possible answers, then explains which answer is correct and why	#content
4) Scientific dissemination	Video explaining the "French paradox" and why it does not fit with today's mainstream nutritional theory, even though it is still used to justify recommending drinking wine with meals	#complementary

As the TikTok account was a private account, all content uploaded to the platform had to be produced by the teacher responsible, as the tools for interacting with other videos on the platform were disabled. However, no specific guidelines were established for the presentation format. This left it up to the teacher to decide on aspects such as which video editing tools to use, the length of the video, and whether or not their face would appear, according to the content to be created.

In order to participate in the project, students were required to register on the TikTok platform by creating a private account. Although registration on the platform was not compulsory, the mark assigned to this project accounted for 10% of the final mark for the module. To obtain the maximum score in this section, students were asked to interact with the TikTok platform, mainly through the *comments* and *likes* tools, and to match their username on the platform with their real name.

To evaluate the project, we designed a quantitative and qualitative analysis framework based on indicators encompassing participating agents, both students and teaching staff. The participating teachers recorded in a session diary any difficulties they experienced in implementing course content through the platform, additional uses that had not yet been considered, and any inconveniences encountered. This feedback was shared in sessions held every 3 weeks. At the end of the study, the students were asked to complete the self-administered voluntary questionnaire described in the following section.

### Sample, questionnaire and data collection

Nursing degree students taking the Psychosocial Sciences Applied to Nursing (PSAN, n = 79) and students of Ageing Nursing (AN, n = 86) modules at the University of Extremadura and Nutrition and Dietetics (ND, n = 48) module at the University of Castilla-La Mancha participated in this study. These modules were chosen due in part to the interest of the teachers in exploring the application of the social media platform, but also due to the fact that there was no overlap of students between these subjects, i.e., each participant in the study was only exposed to one learning environment.

Students completed a self-reported questionnaire, which was made available through a secure virtual platform (Microsoft Forms, Office 365) for one week (16th–22nd May 2022). Participation in the study was voluntary and students received information about the goals of the study and data protection regulations. To answer the questionnaire, it was necessary for the student to consent to participate in the study.

Finally, a questionnaire was developed to assess the students' perception of their experience. This consisted of a first section designed to collect socio-demographic data about the users and a second section designed to capture their assessment of their experiences of using the social media platform as part of their learning. The latter section, in turn, contained qualitative and quantitative questions. The qualitative part of the questionnaire was inspired by the work of Constantinides and colleagues (Constantinides et al., 2013), while the qualitative part was developed following the work of Sprenger and Schwaninger, who had previously adapted the Technology Acceptance Model (TAM) form to digital learning tools (Sprenger & Schwaninger, 2021).

The TAM questionnaires (originally described by Davis (Davis, 1989)) are divided into three parts: one dedicated to measuring perceived usefulness (PU), another to measuring perceived ease of use (PEOU) (after using the tools), and a third part with which the model can predict the behavioural intention (BI) to use the tool in the future. In our questionnaire, the first 5 questions focused on measuring PU, questions 6 to 10 on measuring PEOU, and questions 11 to 15 on measuring BI.

The questionnaire used is included as Appendix A. The first part used a 5-point Likert scale, ranging from 1 (complete disagreement) to 5 (complete agreement), while the TAM questionnaire used the 7-point Likert scale more commonly employed in this type of assessment, with the scale ranging from 1 (complete disagreement) to 7 (complete agreement).

A pilot test was conducted with 10 volunteer students from another subject (Biochemistry, first year) who did not participate in this experience in order to check the consistency of the data obtained and the doubts that students had when filling in the questionnaire.

Finally, the adaptation of the survey from English to Spanish was carried out by our English proofreading service (bilingual Spanish/English), revised by the authors in Spanish and translated back into English and corrected for this publication.

### Ethics statement

In accordance with Declaration of Helsinki guidelines, subjects were informed of the objectives of the study, as well as its anonymous, voluntary, and non-profit nature. Only the researchers involved in the study had access to the collected data.

### Statistical analysis of data

The Kolmogorov–Smirnov test was used to assess the normality of quantitative variables. Where the data did not fit to normality, the nonparametric Mann–Whitney U test was applied to compare between sexes, and the Kruskal–Wallis test to compare between course modules. For qualitative variables, Pearson’s χ^2^ test was used. In all analyses, P values < 0.05 were taken to be statistically significant. Where necessary, post-hoc DMS tests were used to understand differences between groups. Statistical analyses of the data were carried out using version 28.0 of the SPSS statistical package. Qualitative variables have been expressed as percentages, while quantitative variables are presented as the mean ± standard error of the mean (SEM).

## Findings

### Characteristics of the sample

The general characteristics of the sample are presented in Table 2. A total of 213 students responded voluntarily to the questionnaire. All of them agreed to participate in the study. Of the 213 participants, 166 were female (78%) and 47 were male (22%); 123 of them were first-year students (58%), with 81 in their second year (38%), and the remaining 9 further in their studies (4%). The mean age was 20.8 years (0.4 SEM).

**Table 2:** Descriptive analysis of the sample of undergraduate nursing students

	Total (N = 213)	%
Sex		
Male	47	22.1
Female	166	77.9
Age (years)		
	20.8 ± 0.4	
University level		
1st year	123	57.7
2nd year	81	38
3rd year	8	3.8
4th year	1	0.5
Topic		
PSAN	79	37.1
AN	86	40.4
ND	48	22.5

Results are expressed as percentages or as mean ± SEM when indicated. PSAN: Psychosocial Sciences Applied to Nursing; AN: Ageing Nursing; ND: Nutrition and Dietetics.

### Evaluation of the microlearning environment

**Fig. 2:**
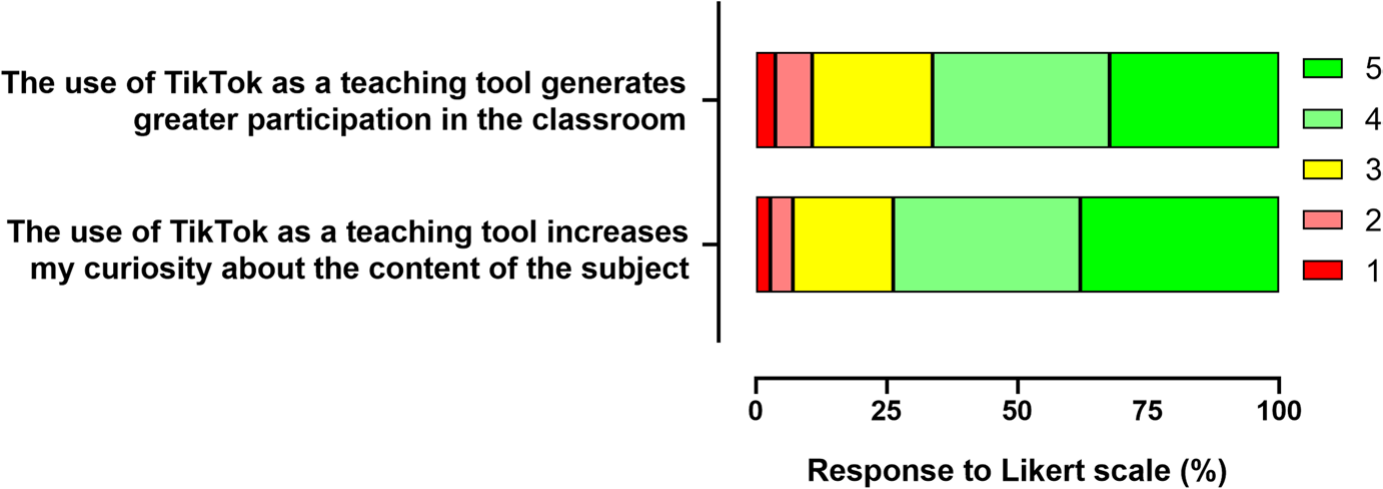
Perceived influence on class participation and willingness to learn more

When users were asked about their perception of the environment created in TikTok as a tool capable of encouraging participation or increasing curiosity about the syllabus content, the results were very positive: for responses given on a scale between 1 and 5, where 1 represents strong disagreement and 5 strong agreement, the average for the first question was 3.84 ± 0.07 and the average for the second question was 4.02 ± 0.07. The distribution of responses is shown in Fig. 2.

**Fig. 3:**
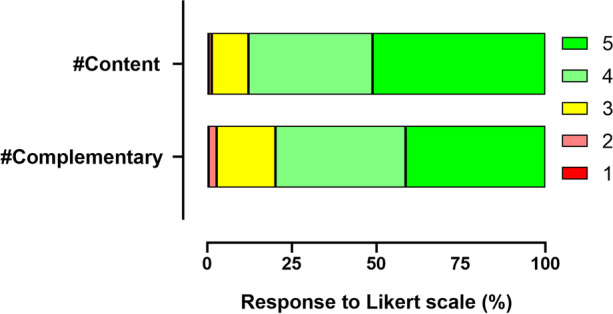
Evaluation of the types of content available on the platform

The students' rating of the two types of content provided through the learning environment was also positive, with an average rating of 4.37 ± 0.05 for content videos (#content) and 4.18 ± 0.06 for complementary content videos (#complementary). The distribution of responses is shown in Fig. 3. These results, together with those presented in Fig. 2, suggest a high degree of participant satisfaction with the experience.

### Assessment of the acceptance of TikTok as a learning tool

**Fig. 4:**
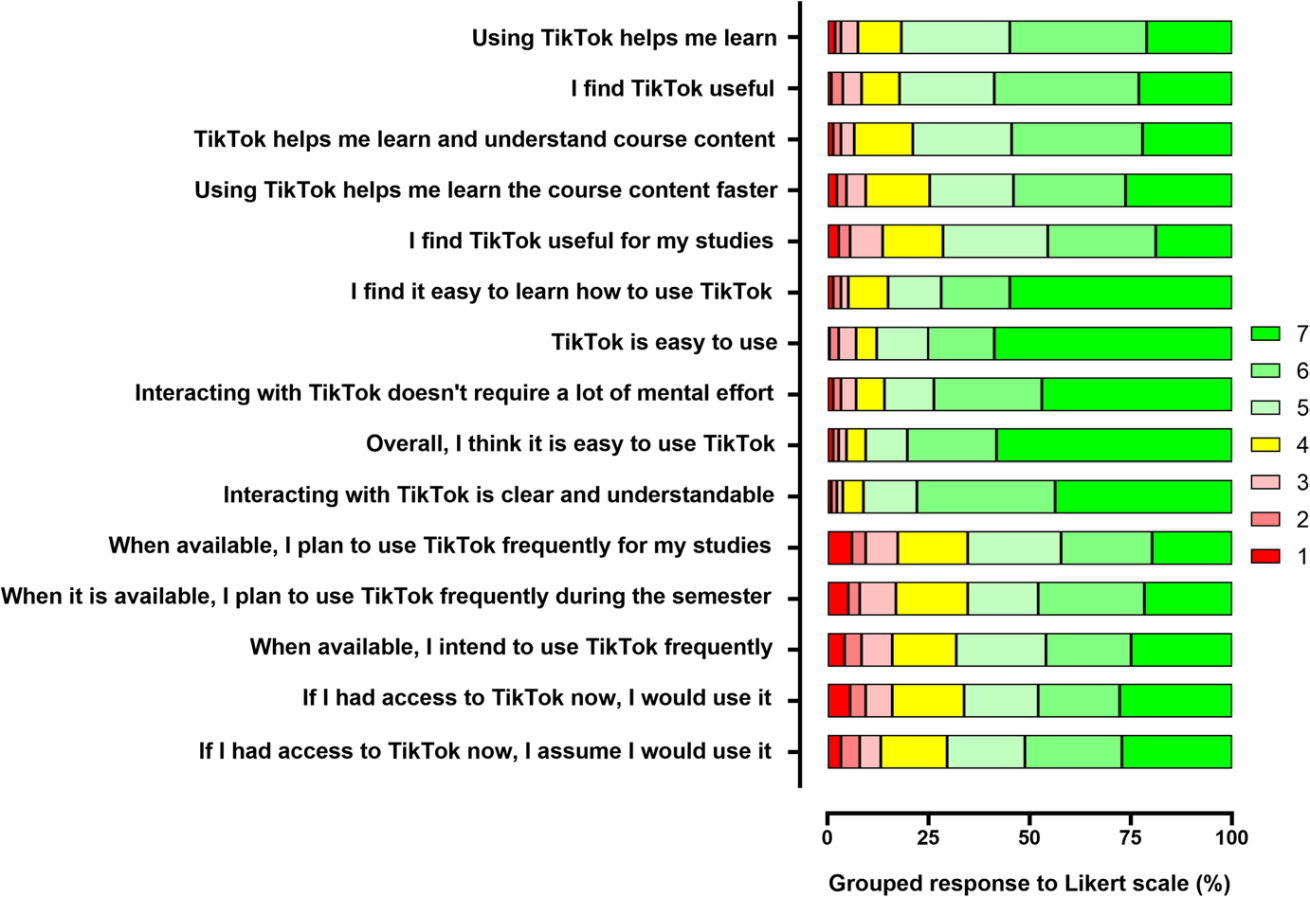
Participants’ responses to the Technology Acceptance Model questionnaire

Acceptance of the use of the TikTok learning environment was assessed by means of a Technology Acceptance Model (TAM) questionnaire, a tool which has previously been used in the literature to measure the degree of acceptance of technologies in the teaching environment. The results obtained are shown in Fig. 4. In general terms, the acceptance of the technology was positive, since none of the 15 items had an average response of less than 5 (with 4 being a neutral score).

**Fig. 5:**
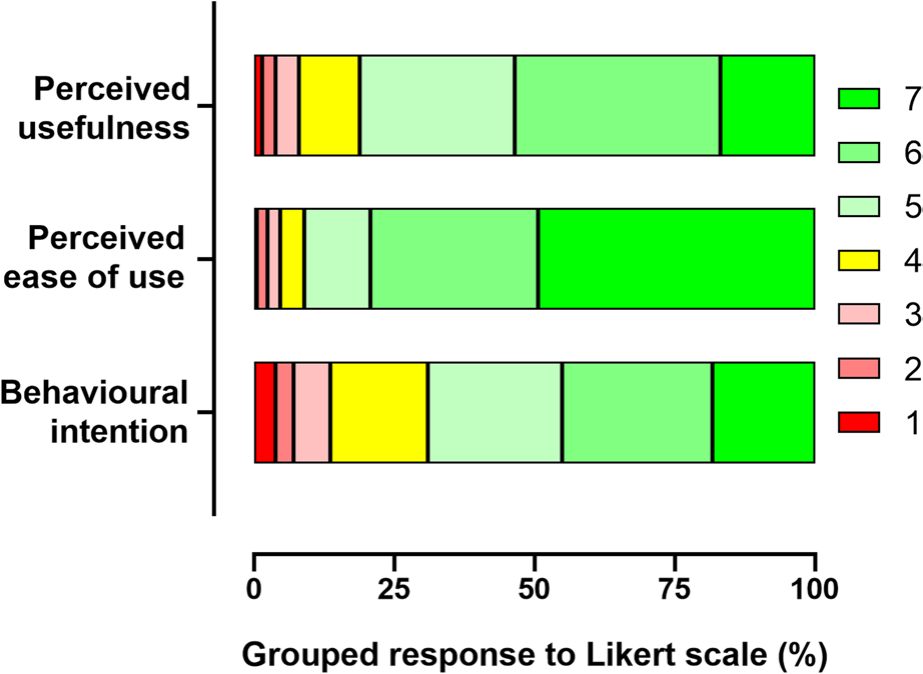
Constructs of the Technology Acceptance Model (TAM)

Based on these results, we proceeded to evaluate user acceptance of the technology by calculating the PU, PEOU and BI, as described in the Methodology section. The results are shown in Fig. 5. The mean score obtained for PU was 5.38 ± 0.08, for PEOU it was 6.07 ± 0.08 and for BI it was 5.09 ± 0.10.

### Influence of the sex of participants on the evaluation of the study

We also studied the influence of the students' sex on their evaluation of the experience. To this end, a statistical analysis of the quantitative and qualitative variables was performed, as described in the Methodology section (results are included in the supplementary file, Tables S1 to S4).

However, it should be noted that there were significant differences (p = 0.022) in the average age of the participants according to sex, with females being younger than males, and that statistically significant (p = 0.044) differences were found in the distribution of sexes between the PSAN and ND subjects, the latter having a much higher female-to-male ratio.

Despite the differences in gender distribution in our sample, most of the variables studied showed no significant difference in the evaluation of the experience by the two sexes.

**Fig. 6:**
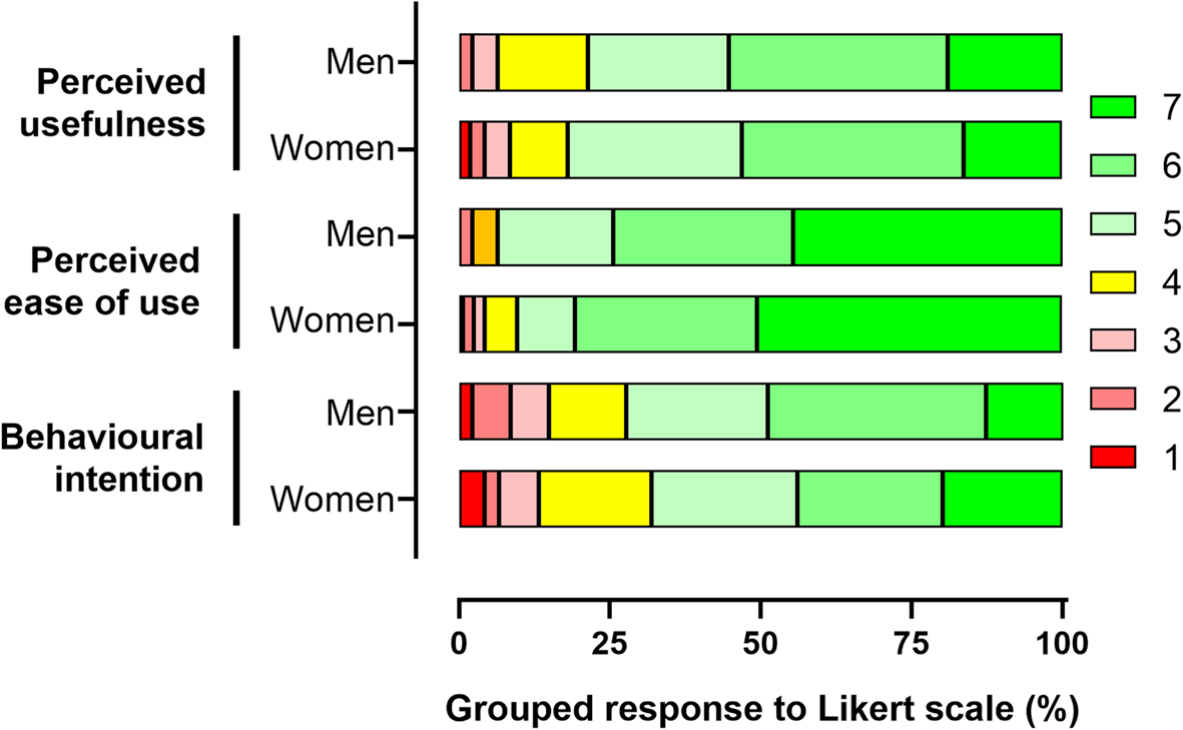
Gender distribution

Accordingly, when we proceeded to evaluate users’ acceptance of this technology by calculating PU, PEOU and BI scores taking participant gender into account, no significant differences were observed, as presented in Fig. 6. These results suggest that the sex of the participants has little influence on their assessment of this pedagogical experience.

### Influence of the module in which the participants were enrolled on the evaluation of the experience

Finally, the extent to which the subject in which each microlearning environment was implemented influenced the students' assessment was analysed. For this purpose, a statistical analysis of the qualitative and qualitative variables obtained was performed, as described in the Methodology section (results are included in the supplementary file, Tables S5 to S8). In contrast to the influence of participant gender, the module in which the environment was implemented appears to have a more marked influence on the students' assessment of the teaching experience. In fact, significant differences were found in virtually all aspects of the microenvironment assessment when the module being taken by the participants was included in the analysis.

In terms of the participants' perception of the influence of the use of TikTok on increasing their participation in class, students reported greater increases in participation in the PSAN and ND modules, while students’ reported increase in participation in the AN module was lower. A similar situation was observed when analysing the data on the participants' perception of the increase in their curiosity, where again students taking the PSAN and ND modules reported higher increases than for AN. Finally, this trend is also reflected in the results of the analysis based on the nature of the videos uploaded to the platform. It is, however, important to highlight that the assessments were positive in all cases (the overall average of the assessments was higher than 3, which is the neutral score on the 5-point Likert scale employed, while the averages for the PSAN and ND modules were above 4 in all cases).

**Fig. 7:**
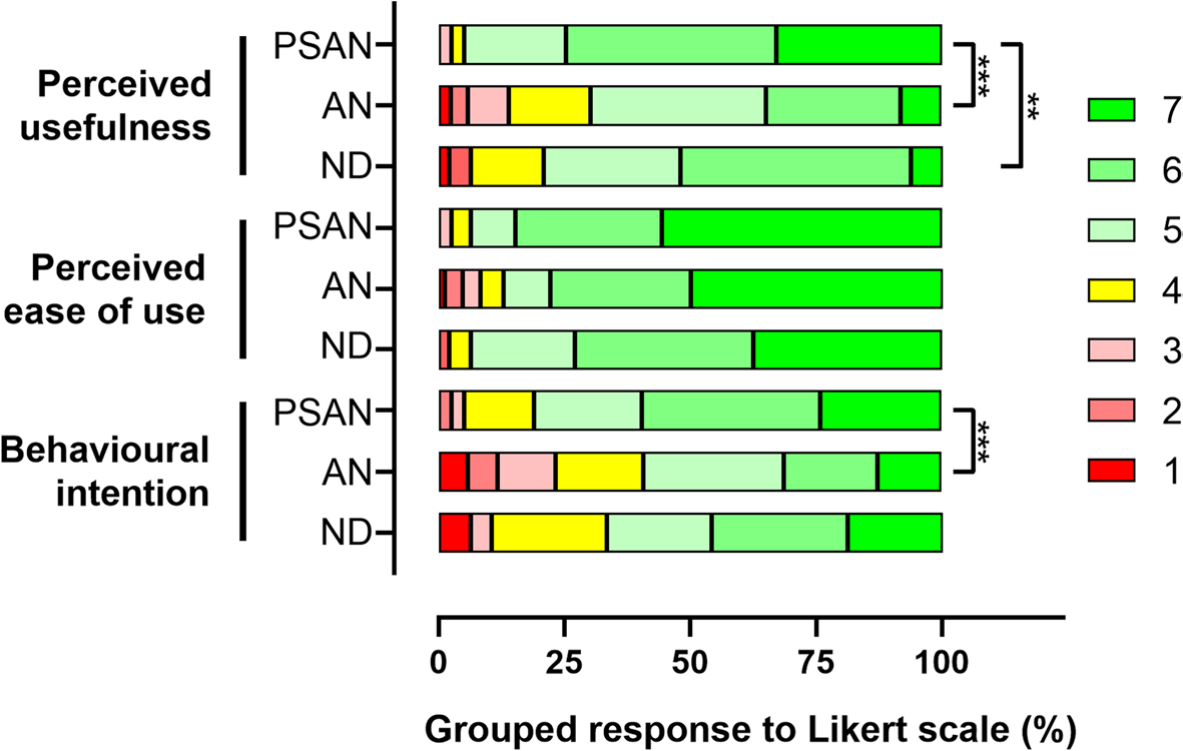
Subject distribution

As for the previous analyses, the TAM responses were used to calculate the PU, PEOU and BI scores, differentiating between the teaching module in which the microlearning environment was implemented. The analysis of the data shows differences in both PU and BI, with the PSAN module obtaining the highest scores in both cases, while no differences were observed for the PEOU construct. Similarly to the results reported above, the participants' assessments broken down by the module they were taking continue to be positive (on a 7-point Likert scale, the mean is above 4, which is the neutral score, and above 5 in all cases for the PSAN and ND modules). These results are shown in Fig. 7.

However, the observed differences in the evaluations of the microlearning environments are not significant enough to alter the clearly positive assessment of the experience by the students, even in the most unfavourable case. This suggests that the differences intrinsic to the design of the experiment (for example, the teacher responsible for each participating module or the topics covered) are not sufficient to cause participants to reject the model.

## Discusion

This paper evaluates the possibilities and limitations of introducing TikTok-based microlearning to university-level health sciences teaching. Our results presented above for a sample of students enrolled in three different modules of the bachelor’s degree in Nursing (N = 213) demonstrate a broad acceptance of the experience. However, when the acceptance of the environment was analysed according to the TAM model, differences were observed in the participants' assessment depending on the environment (the academic module) in which they were exposed to the learning environment. The participants' assessments were independent of other factors (such as gender). These subtle differences may be due to various factors: the year of study in which the teaching is offered (first or second year), contrasts with the pedagogical model of the other subjects being taught in the same semester, the predisposition of each group of students towards teaching innovation, and even the influence of the individual teachers (for example, if a certain teacher provided more detail when developing the microlearning content). Furthermore, although the activity was designed to use a similar structure across each environment, the content and activities specific to each subject may have influenced the way in which the participants perceived the experience.

Given that the application we worked with is ready to use (as well as being widely adopted), we expected to—and indeed did—find high values for the PEOU scores. High scores for PU and BI were also to be expected since the microcontent offered through the platform provides help to pass the course and was therefore aligned with the students’ objectives (Biggs, 2003). One obvious factor that may have influenced these positive ratings is the fact that we chose to develop our learning environments using an application that is so popular with students.

Our study has, however, yielded a surprising result in that, despite the consensus in the literature which assumes that anything that does not provide exam-relevant input will not be aligned with the students' interests, and will therefore be less valued (FitzPatrick et al., 2015), our participants positively valued the fact that the experience has helped them to increase their curiosity towards the subject content. This is reflected in the fact that the participants evaluated the videos on complementary content just as positively (with no statistically significant differences) as the videos with exam-related content.

In our opinion, one of the strong points of using TikTok to develop learning environments is the simplicity that it provides when creating content, as one is not limited to relying on available content created by other users (one of the classic limitations of virtual learning environments). Instead, the teacher can easily create their own content following a homogeneous format with respect to the rest of the content they upload to the platform, which in turn makes it easier for the end users to accept and use the technology.

The original design of the project emphasised a teacher-only approach to content creation, although some examples of student content creation arose spontaneously during the course of the project, hinting at directions for future developments in peer-to-peer and peer-to-teacher communication. This aligns with the findings of Radin (Radin & Light, 2022), outlining best practices for effective and engaging science communication teaching and learning. Most works using social networks such as Facebook (Legaree, 2014) and Tik Tok itself (Yélamos-Guerra et al., 2022) have reported improvements in student motivation and student engagement with the learning process (Cooke, 2017); this is also reflected in the results of our study, surpassing the results for other types of tools, such as blogs (Conde-Caballero et al., 2019).

Previous studies incorporating qualitative perspectives (Gallardo Echenique et al., 2015; Smith, 2017) have noted that social networks became important to the extent that they served as a means of communication and a way of staying "up to date". TikTok can be employed as a means of communication with the student group, although it does not seem to improve on more conventional channels such as email or Moodle. It has also been reported that students use social media in their own learning, and so developing students' knowledge and skills in terms of wider digital literacy could foster their ability to integrate the beneficial aspects of social media that support their learning, while also mitigating the pitfalls that can hinder learning (Smith, 2016). It is also interesting to note that students' use of the tool tends to centre more on content consumption than creation.

Some limitations associated with the use of TikTok to promote microlearning cannot be ignored: some authors have pointed out that the lower reliability of student-produced materials not subject to peer review could lead to confusing knowledge processes (Ovaere et al., 2018). In our work, the greater focus on teacher-created content means that fewer errors can be assumed. On the other hand, with teacher supervision, it may even be possible to take advantage of this phenomenon to deepen the critical processes of generating and constructing evidence. Other works warn of the danger of focusing education on tools or media, which may in fact increase the separation between teacher and student by altering the final objective of such a relationship (Yancey, 2017). We believe that our work is a productive use of microlearning tools that complement traditional teaching, but are not to be taken as a substitute for it.

### Limitations of the study

As with any study in educational sciences, the present study has certain limitations, which means that the outcomes of this work should be interpreted with caution and contextualised in the respective teaching situation. Firstly, the sample size is relatively small. Therefore, it would be desirable to extend this study both to obtain a larger sample size, and to observe whether there are differences between other subjects in the field of health and care. Furthermore, this study did not include a control group. It is widely recognised that the inclusion of control groups in teaching experiences is not always easy and sometimes raises ethical dilemmas. However, just as it would be desirable to extend these experiences, it would also be useful to compare results against a control group. In addition, the criteria used to define the two main categories to which the produced content was uploaded were chosen ad hoc, so it would be interesting to contrast other proposals that use different terminologies to categorise the content. Finally, there are differences intrinsic to each teacher, group of students and subject matter that may influence how these microlearning environments are developed and implemented, and thus how they are evaluated by the students.

### Future perspectives

As explored in the previous paragraph, further studies are needed to explore the full potential of these TikTok-generated microlearning environments in the field of health sciences. These studies should not only focus on the teaching possibilities offered by the platform (i.e., the possibility of generating content and sharing it in a learning environment), but also on understanding the factors that favour students’ acceptance one type of technology over another. After all, it is tempting to assume that the technology that is more easily accepted should be the one that promotes more learning among our students. For this reason, it would be very interesting to obtain sufficient evidence (e.g., by measuring students' academic performance) to demonstrate which types of tools or teaching interventions would be the most appropriate when it comes to promoting high-quality learning through microlearning environments in a university population.

## Conclusions

The outcomes of this study reinforce our hypothesis that justifies the application of social networks as a complementary tool in university teaching, especially in health sciences. The students' assessment of the teaching experience is clearly positive in the areas analysed: engagement with the different subjects, motivation for the mandatory and complementary content and competences, as well as the high level of acceptance of the technology in the different constructs of the TAM. On the other hand, no significant differences were observed in terms of the evaluations of the experience according to gender, but significant differences were observed according to the subject in which the microlearning environment was deployed. Overall, our work shows very positive evaluations by students when the TikTok platform was used to promote high-quality learning through microlearning.

### Electronic supplementary material

Below is the link to the electronic supplementary material.

Supplementary file1 (DOCX 86.7 KB)

## Data Availability

To ensure students’ privacy, the datasets analysed in the present study are not publicly available. They are, however, are available from the corresponding author on reasonable request.

## Appendix A

- Part A: Socio-demographic characteristics.
  1. Sex
    - Man
    - Woman
    - Other
  2. Age (years)
  3. Current year of university studies
    - First
    - Second
    - …
  4. From where do you mainly access the platform?
    - Home network
    - University network
    - Mobile network
    - Other
- Part B: Rating of the perceived use of the TikTok tool.
  1. Please rate the following statements between 1 and 5, where 1 represents strong disagreement and 5 represents strong agreement.
    - The use of TikTok as a teaching tool promotes greater participation in the classroom
    - The use of TikTok as a teaching tool increases my curiosity about the content of the module
  2. How would you rate the review content videos? (from 1 to 5 stars)
  3. How would you rate the complementary content videos? (from 1 to 5 stars)
- Part C: Assessment of the acceptance of TikTok as a learning tool.
  Please read the following statements carefully and indicate how much you agree with them on a scale of 1 to 7, where 1 represents strong disagreement, 7 represents strong agreement, and 4 indicates that you neither agree nor disagree:
  1. Using TikTok helps me learn
  2. I find TikTok useful
  3. TikTok helps me learn and understand course content
  4. Using TikTok helps me learn the course content faster
  5. I find TikTok useful for my studies
  6. I find it easy to learn how to use TikTok
  7. TikTok is easy to use
  8. Interacting with TikTok doesn't require a lot of mental effort
  9. Overall, I think it is easy to use TikTok
  10. Interacting with TikTok is clear and understandable
  11. When it is available, I plan to use TikTok frequently for my studies
  12. When it is available, I plan to use TikTok frequently during the semester
  13. When it is available, I intend to use TikTok frequently
  14. If I had access to TikTok now, I would use it
  15. If I had access to TikTok now, I assume I would use it
