# Supplementary material for: Microlearning through TikTok in Higher Education. An evaluation of uses and potentials
Source: Educ Inf Technol (Dordr). 2023 Jun 2:1–21. Online ahead of print. doi: 10.1007/s10639-023-11904-4 (PMC10235824; doi:10.1007/s10639-023-11904-4)
Supplement: Supplementary file 1 — Supplementary file1 (DOCX 86.7 KB) [file 10639_2023_11904_MOESM1_ESM.docx]

**Table S.1.** Classification of study participants according to sex. Results are expressed as percentages or as mean ± SEM when indicated. The significance of differences according to sex was determined as indicated in Methodology for quantitative and qualitative variables. Statistically significant results are highlighted in bold.

|  | **Men** | **%** | **Woman** | **%** | **p-value** | **Post hoc  DMS Test** |
| --- | --- | --- | --- | --- | --- | --- |
| **Population** |  |  |  |  |  |  |
|  | 47 | 22.1 | 166 | 77.9 |  |  |
| **Age (years)** |  |  |  |  |  |  |
|  | 24.2 ± 1.6 |  | 19.8 ± 0.3 |  | **0.022** |  |
| **University level** |  |  |  |  |  |  |
| **1st year** | 23 | 55.3 | 97 | 58.4 | 0.778 |  |
| **2nd year** | 20 | 42.6 | 61 | 36.7 |  |  |
| **3rd year** | 1 | 2.1 | 7 | 4.2 |  |  |
| **4th year** | 0 | 0.0 | 1 | 0.6 |  |  |
| **Topic** |  |  |  |  | **0.049** |  |
| **PSAN** | 22 | 47.0 | 57 | 34.3 |  | PSAN vs ND **0.044** |
| **AN** | 19 | 40.0 | 67 | 40.4 |  | AN vs PSAN 0.373 |
| **ND** | 6 | 13.0 | 42 | 25.3 |  | ND vs AN 0.438 |

**Table S.2.** Gender distribution of students’ responses on the perceived influence of TikTok use on class participation and willingness to learn more. N=213.

|  | **Strongly disagree** | **%** | **Disagree** | **%** | **Neither agree nor disagree** | **%** | **Agree** | **%** | **Strongly agree** | **%** | **p-value** |
| --- | --- | --- | --- | --- | --- | --- | --- | --- | --- | --- | --- |
| **The use of TikTok as a teaching tool generates greater participation in the classroom** |  |  |  |  |  |  |  |  |  |  | 0.340 |
| **Men** | 2 | 4.3 | 6 | 12.8 | 12 | 25.5 | 16 | 34.0 | 11 | 23.4 |  |
| **Women** | 6 | 3.6 | 9 | 5.4 | 37 | 22.3 | 56 | 33.7 | 58 | 34.9 |  |
| **The use of TikTok as a teaching tool increases my curiosity about the content of the subject** |  |  |  |  |  |  |  |  |  |  | 0.927 |
| **Men** | 1 | 2.1 | 2 | 4.2 | 11 | 23.4 | 15 | 31.9 | 18 | 38.3 |  |
| **Women** | 5 | 3.0 | 7 | 4.2 | 30 | 18.1 | 61 | 26.7 | 63 | 38.0 |  |

**Table S.3.** Gender distribution of participants' responses on their evaluation of the two main types of content available on the platform. N=213.

|  | **Strongly disagree** | **%** | **Disagree** | **%** | **Neither agree nor disagree** | **%** | **Agree** | **%** | **Strongly agree** | **%** | **p-value** |
| --- | --- | --- | --- | --- | --- | --- | --- | --- | --- | --- | --- |
| **#Content** |  |  |  |  |  |  |  |  |  |  | 0.659 |
| **Men** | 0 | 0 | 0 | 0.0 | 5 | 10.6 | 21 | 44.7 | 21 | 44.7 |  |
| **Women** | 1 | 0.6 | 2 | 1.2 | 18 | 10.8 | 57 | 34. | 88 | 53.0 |  |
| **#Complementary** |  |  |  |  |  |  |  |  |  |  | 0.463 |
| **Men** | 0 | 0.0 | 0 | 0.0 | 10 | 21.3 | 21 | 44.7 | 16 | 34.0 |  |
| **Women** | 1 | 0.6 | 5 | 3.0 | 27 | 16.3 | 61 | 36.7 | 72 | 43.4 |  |

**Table S.4.** Gender distribution of results obtained for each of the 15 answers to the Technology Acceptance Model questionnaire. Statistically significant results are highlighted in bold. N=213.

|  | **Strongly disagree** | **%** | **Disagree** | **%** | **Slightly disagree** | **%** | **Neither agree nor disagree** | **%** | **Slightly agree** | **%** | **Agree** | **%** | **Strongly agree** | **%** | **p-value** |
| --- | --- | --- | --- | --- | --- | --- | --- | --- | --- | --- | --- | --- | --- | --- | --- |
| **Q1** |  |  |  |  |  |  |  |  |  |  |  |  |  |  | 0.924 |
| **Men** | 1 | 2.1 | 0 | 0 | 2 | 4.3 | 5 | 10.6 | 15 | 31.9 | 16 | 34.0 | 8 | 17.0 |  |
| **Women** | 3 | 1.8 | 3 | 1.8 | 7 | 4.2 | 18 | 10.8 | 42 | 25.3 | 56 | 33.7 | 37 | 23.3 |  |
| **Q2** |  |  |  |  |  |  |  |  |  |  |  |  |  |  | 0.627 |
| **Men** | 0 | 0 | 0 | 0 | 2 | 4.3 | 7 | 14.9 | 10 | 21.3 | 17 | 36.2 | 11 | 23.4 |  |
| **Women** | 2 | 1.2 | 6 | 3.6 | 8 | 4.8 | 13 | 7.8 | 40 | 24.1 | 59 | 35.5 | 38 | 22.9 |  |
| **Q3** |  |  |  |  |  |  |  |  |  |  |  |  |  |  | 0.671 |
| **Men** | 0 | 0.0 | 0 | 0.0 | 2 | 4.3 | 8 | 17.0 | 14 | 29.8 | 12 | 25.5 | 11 | 23.4 |  |
| **Women** | 3 | 1.8 | 4 | 2.4 | 5 | 3.0 | 23 | 13.9 | 38 | 22.9 | 57 | 34.3 | 36 | 21.7 |  |
| **Q4** |  |  |  |  |  |  |  |  |  |  |  |  |  |  | 0.704 |
| **Men** | 1 | 2.1 | 0 | 0 | 1 | 2.1 | 9 | 19.1 | 12 | 25.5 | 11 | 23.4 | 13 | 27.7 |  |
| **Women** | 4 | 2.4 | 5 | 3.0 | 9 | 5.4 | 25 | 15.1 | 32 | 19.3 | 48 | 28.9 | 43 | 25.9 |  |
| **Q5** |  |  |  |  |  |  |  |  |  |  |  |  |  |  | 0.505 |
| **Men** | 0 | 0.0 | 2 | 4.3 | 6 | 12.8 | 8 | 17.0 | 9 | 19.1 | 12 | 25.5 | 10 | 21.3 |  |
| **Women** | 6 | 3.6 | 4 | 2.4 | 11 | 6.6 | 24 | 14.5 | 46 | 27.7 | 45 | 27.1 | 30 | 18.1 |  |
| **Q6** |  |  |  |  |  |  |  |  |  |  |  |  |  |  | 0.702 |
| **Men** | 0 | 0.0 | 2 | 4.3 | 1 | 2.1 | 3 | 6.4 | 6 | 12.8 | 7 | 14.9 | 28 | 59.6 |  |
| **Women** | 3 | 1.8 | 2 | 1.2 | 3 | 1.8 | 18 | 10.8 | 22 | 13.3 | 29 | 17.5 | 89 | 53.6 |  |
| **Q7** |  |  |  |  |  |  |  |  |  |  |  |  |  |  | 0.278 |
| **Men** | 0 | 0.0 | 2 | 4.3 | 1 | 2.1 | 1 | 2.1 | 9 | 19.1 | 4 | 8.5 | 30 | 63.8 |  |
| **Women** | 1 | 0.6 | 3 | 1.8 | 8 | 4.8 | 10 | 6.0 | 18 | 10.8 | 31 | 18.7 | 95 | 57.2 |  |
| **Q8** |  |  |  |  |  |  |  |  |  |  |  |  |  |  | 0.868 |
| **Men** | 0 | 0.0 | 1 | 2.1 | 1 | 2.1 | 5 | 10.6 | 6 | 12.8 | 13 | 27.7 | 21 | 44.7 |  |
| **Women** | 3 | 1.8 | 3 | 1.8 | 7 | 4.2 | 10 | 6.0 | 20 | 12.0 | 44 | 26.5 | 79 | 17.6 |  |
| **Q9** |  |  |  |  |  |  |  |  |  |  |  |  |  |  | 0.587 |
| **Men** | 0 | 0.0 | 1 | 2.1 | 2 | 4.3 | 1 | 2.1 | 6 | 12.8 | 12 | 25.5 | 25 | 53.2 |  |
| **Women** | 3 | 1.8 | 2 | 1.2 | 2 | 1.2 | 9 | 5.4 | 16 | 9.6 | 35 | 21.1 | 99 | 59.6 |  |
| **Q10** |  |  |  |  |  |  |  |  |  |  |  |  |  |  | 0.519 |
| **Men** | 0 | 0.0 | 1 | 2.1 | 0 | 0.0 | 4 | 8.5 | 8 | 17.0 | 18 | 38.3 | 16 | 34.0 |  |
| **Women** | 2 | 1.2 | 2 | 1.2 | 3 | 1.8 | 7 | 4.2 | 20 | 12.0 | 55 | 33.1 | 77 | 46.4 |  |
| **Q11** |  |  |  |  |  |  |  |  |  |  |  |  |  |  | 0.607 |
| **Men** | 4 | 8.5 | 1 | 2.1 | 2 | 4.3 | 7 | 14.9 | 14 | 29.8 | 8 | 17.0 | 11 | 23.4 |  |
| **Women** | 9 | 5.4 | 6 | 3.6 | 15 | 9.0 | 30 | 18.1 | 35 | 21.1 | 40 | 24.1 | 31 | 18.7 |  |
| **Q12** |  |  |  |  |  |  |  |  |  |  |  |  |  |  | 0.666 |
| **Men** | 3 | 6.4 | 2 | 4.3 | 2 | 4.3 | 8 | 17.0 | 8 | 17.0 | 16 | 34.0 | 8 | 17.0 |  |
| **Women** | 8 | 4.8 | 4 | 2.4 | 17 | 10.2 | 30 | 18.1 | 29 | 17.5 | 40 | 24.1 | 38 | 22.9 |  |
| **Q13** |  |  |  |  |  |  |  |  |  |  |  |  |  |  | **0.047** |
| **Men** | 2 | 4.3 | 3 | 6.4 | 3 | 6.4 | 4 | 8.5 | 8 | 17.0 | 18 | 38.3 | 9 | 19.1 |  |
| **Women** | 7 | 4.2 | 6 | 3.6 | 13 | 7.8 | 30 | 18.1 | 39 | 23.5 | 27 | 16.3 | 44 | 26.5 |  |
| **Q14** |  |  |  |  |  |  |  |  |  |  |  |  |  |  | 0.449 |
| **Men** | 3 | 6.4 | 3 | 6.4 | 1 | 2.1 | 9 | 19.1 | 10 | 21.3 | 12 | 25.5 | 9 | 19.1 |  |
| **Women** | 9 | 5.4 | 5 | 3.0 | 13 | 7.8 | 29 | 17.5 | 29 | 17.5 | 31 | 18.7 | 50 | 30.1 |  |
| **Q15** |  |  |  |  |  |  |  |  |  |  |  |  |  |  | 0.111 |
| **Men** | 1 | 2.1 | 5 | 10.6 | 0 | 0.0 | 8 | 17.0 | 10 | 21.3 | 14 | 29.8 | 9 | 19.1 |  |
| **Women** | 6 | 3.6 | 5 | 3.0 | 11 | 6.6 | 27 | 16.3 | 31 | 18.7 | 37 | 22.3 | 49 | 29.5 |  |

**Table S.5.** Classification of the study participants according to the subject they were enrolled in. Results are expressed as percentages or as mean ± SEM when indicated. Statistically significant results are highlighted in bold and were determined as indicated in Methodology for quantitative and qualitative variables.

|  | **PSAN** | **%** | **AN** | **%** | **ND** | **%** | **p-value** |
| --- | --- | --- | --- | --- | --- | --- | --- |
| **Population** |  |  |  |  |  |  |  |
|  | 79 | 37.2 | 86 | 40.3 | 48 | 22.5 |  |
| **Age (years)** |  |  |  |  |  |  | 0.541 |
|  | 20.3 ± 0.8 |  | 21.3 ± 0.6 |  | 20.6 ± 0.9 |  |  |
| **University level** |  |  |  |  |  |  | **<0.001** |
| **1st year** | 76 | 96.2 |  |  | 47 | 97.9 |  |
| **2nd year** | 3 | 3.8 | 77 | 89.5 | 1 | 2.1 |  |
| **3rd year** |  |  | 8 | 9.4 |  |  |  |
| **4th year** |  |  | 1 | 0.1 |  |  |  |

PSAN: Psychosocial Sciences Applied to Nursing; AN: Ageing Nursing; ND: Nutrition and Dietetics.

**Table S.6.** Distribution of students’ responses on the perceived influence of TikTok use on class participation and willingness to learn more according to the subjects attended by the participants. Statistically significant results are highlighted in bold. N=213.

|  | **Strongly disagree** | **%** | **Disagree** | **%** | **Neither agree nor disagree** | **%** | **Agree** | **%** | **Strongly agree** | **%** | **p-value** |
| --- | --- | --- | --- | --- | --- | --- | --- | --- | --- | --- | --- |
| **The use of TikTok as a teaching tool generates greater participation in the classroom** |  |  |  |  |  |  |  |  |  |  | **<0.001** |
| **PSAN** | 1 | 1.3 | 1 | 1.3 | 18 | 22.8 | 29 | 36.7 | 30 | 38.0 |  |
| **AN** | 6 | 7.0 | 13 | 15.1 | 27 | 31.4 | 26 | 30.2 | 14 | 16.3 |  |
| **ND** | 1 | 2.1 | 1 | 2.1 | 4 | 8.3 | 17 | 35.4 | 25 | 52.1 |  |
| **The use of TikTok as a teaching tool increases my curiosity about the content of the subject** |  |  |  |  |  |  |  |  |  |  | **<0.001** |
| **PSAN** | 0 | 0.0 | 1 | 1.3 | 12 | 15.2 | 26 | 32.9 | 40 | 50.6 |  |
| **AN** | 5 | 5.8 | 7 | 8.1 | 26 | 30.2 | 30 | 34.9 | 18 | 20.9 |  |
| **ND** | 1 | 2.1 | 1 | 2.1 | 3 | 6.3 | 20 | 41.7 | 23 | 47.9 |  |

PSAN: Psychosocial Sciences Applied to Nursing; AN: Ageing Nursing; ND: Nutrition and Dietetics.

**Table S.7.** Distribution of responses on students’ evaluation of the two main types of content available on the platform according to the subjects attended by the participants. Statistically significant results are highlighted in bold. N=213.

|  | **Strongly disagree** | **%** | **Disagree** | **%** | **Neither agree nor disagree** | **%** | **Agree** | **%** | **Strongly agree** | **%** | **p-value** |
| --- | --- | --- | --- | --- | --- | --- | --- | --- | --- | --- | --- |
| **#Content** |  |  |  |  |  |  |  |  |  |  | **<0.001** |
| **PSAN** | 0 | 0.0 | 0 | 0.0 | 1 | 1.3 | 20 | 25.3 | 58 | 73.4 |  |
| **AN** | 1 | 1.2 | 1 | 1.2 | 16 | 18.6 | 39 | 45.3 | 29 | 33.7 |  |
| **ND** | 0 | 0.0 | 1 | 2.1 | 6 | 12.5 | 19 | 39.6 | 22 | 45.8 |  |
| **#Complementary** |  |  |  |  |  |  |  |  |  |  | **0.036** |
| **PSAN** | 0 | 0.0 | 0 | 0.0 | 12 | 15.2 | 25 | 31.6 | 42 | 53.2 |  |
| **AN** | 1 | 1.2 | 4 | 4.7 | 18 | 20.9 | 40 | 46.5 | 23 | 26.7 |  |
| **ND** | 0 | 0.0 | 1 | 2.1 | 7 | 14.6 | 17 | 35.4 | 23 | 47.9 |  |

PSAN: Psychosocial Sciences Applied to Nursing; AN: Ageing Nursing; ND: Nutrition and Dietetics.

**Table S.8.** Distribution of results obtained for each of the 15 answers to the Technology Acceptance Model questionnaire according to the subjects attended by the participants. Statistically significant results are highlighted in bold. N=213.

|  | **Strongly disagree** | **%** | **Disagree** | **%** | **Slightly disagree** | **%** | **Neither agree nor disagree** | **%** | **Slightly agree** | **%** | **Agree** | **%** | **Strongly agree** | **%** | **p-value** |
| --- | --- | --- | --- | --- | --- | --- | --- | --- | --- | --- | --- | --- | --- | --- | --- |
| **Q1** |  |  |  |  |  |  |  |  |  |  |  |  |  |  | **0.014** |
| **PSAN** | 1 | 1.3 | 0 | 0.0 | 1 | 1.3 | 5 | 6.3 | 18 | 22.8 | 29 | 36.7 | 25 | 31.6 |  |
| **AN** | 2 | 2.3 | 2 | 2.3 | 6 | 7.0 | 15 | 17.4 | 26 | 30.2 | 20 | 23.3 | 15 | 17.4 |  |
| **ND** | 1 | 2.1 | 1 | 2.1 | 2 | 4.2 | 3 | 6.3 | 13 | 27.1 | 23 | 27.9 | 5 | 10.4 |  |
| **Q2** |  |  |  |  |  |  |  |  |  |  |  |  |  |  | 0.130 |
| **PSAN** | 0 | 0.0 | 0 | 0.0 | 1 | 1.3 | 5 | 6.3 | 17 | 21.5 | 32 | 40.5 | 24 | 30.4 |  |
| **AN** | 2 | 2.3 | 4 | 4.7 | 7 | 8.1 | 11 | 12.8 | 21 | 24.4 | 25 | 29.1 | 16 | 18.6 |  |
| **ND** | 0 | 0.0 | 2 | 4.2 | 2 | 4.2 | 4 | 8.3 | 12 | 25.0 | 19 | 29.6 | 9 | 18.8 |  |
| **Q3** |  |  |  |  |  |  |  |  |  |  |  |  |  |  | **<0.001** |
| **PSAN** | 0 | 0.0 | 0 | 0.0 | 1 | 1.3 | 7 | 8.9 | 12 | 15.2 | 26 | 32.9 | 33 | 41.8 |  |
| **AN** | 3 | 3.5 | 1 | 1.2 | 5 | 5.8 | 19 | 22.1 | 26 | 30.2 | 22 | 25.6 | 10 | 11.6 |  |
| **ND** | 0 | 0.0 | 3 | 6.3 | 1 | 2.1 | 5 | 10.4 | 14 | 29.2 | 21 | 43.8 | 4 | 8.3 |  |
| **Q4** |  |  |  |  |  |  |  |  |  |  |  |  |  |  | **<0.001** |
| **PSAN** | 0 | 0.0 | 0 | 0.0 | 1 | 1.3 | 6 | 7.6 | 8 | 10.1 | 25 | 31.6 | 39 | 49.4 |  |
| **AN** | 4 | 4.7 | 4 | 4.7 | 7 | 8.1 | 21 | 24.4 | 21 | 24.4 | 18 | 20.9 | 11 | 12.8 |  |
| **ND** | 1 | 2.1 | 1 | 2.1 | 2 | 4.2 | 7 | 14.6 | 15 | 31.3 | 16 | 33.3 | 6 | 12.5 |  |
| **Q5** |  |  |  |  |  |  |  |  |  |  |  |  |  |  | **0.001** |
| **PSAN** | 0 | 0.0 | 1 | 1.3 | 3 | 3.8 | 8 | 10.1 | 14 | 17.7 | 27 | 34.2 | 26 | 32.9 |  |
| **AN** | 4 | 4.7 | 4 | 4.7 | 10 | 11.6 | 18 | 20.9 | 23 | 26.7 | 17 | 19.8 | 10 | 11.6 |  |
| **ND** | 2 | 4.7 | 1 | 2.1 | 4 | 8.3 | 6 | 12.5 | 18 | 37.5 | 13 | 27.1 | 4 | 8.3 |  |
| **Q6** |  |  |  |  |  |  |  |  |  |  |  |  |  |  | **0.021** |
| **PSAN** | 0 | 0.0 | 0 | 0.0 | 3 | 3.8 | 6 | 7.6 | 4 | 5.1 | 15 | 19.0 | 51 | 64.6 |  |
| **AN** | 2 | 2.3 | 4 | 4.7 | 1 | 1.2 | 8 | 9.3 | 12 | 14.0 | 14 | 16.3 | 45 | 52.3 |  |
| **ND** | 1 | 2.1 | 0 | 0.0 | 0 | 0.0 | 7 | 14.6 | 12 | 25.0 | 7 | 14.6 | 21 | 43.8 |  |
| **Q7** |  |  |  |  |  |  |  |  |  |  |  |  |  |  | 0.143 |
| **PSAN** | 0 | 0.0 | 0 | 0.0 | 4 | 5.1 | 5 | 6.3 | 5 | 3.3 | 14 | 17.7 | 51 | 64.6 |  |
| **AN** | 1 | 1.2 | 4 | 4.7 | 2 | 2.3 | 2 | 2.3 | 13 | 15.1 | 11 | 12.8 | 53 | 61.6 |  |
| **ND** | 0 | 0.0 | 1 | 2.1 | 3 | 6.3 | 4 | 8.3 | 9 | 18.8 | 10 | 20.8 | 21 | 43.8 |  |
| **Q8** |  |  |  |  |  |  |  |  |  |  |  |  |  |  | **0.003** |
| **PSAN** | 0 | 0.0 | 1 | 1.3 | 1 | 1.3 | 12 | 15.2 | 6 | 7.6 | 20 | 25.3 | 39 | 49. |  |
| **AN** | 2 | 2.3 | 3 | 3.5 | 5 | 5.8 | 3 | 3.5 | 11 | 12.8 | 17 | 19.8 | 45 | 52.3 |  |
| **ND** | 1 | 2.1 | 0 | 0.0 | 2 | 4.2 | 0 | 0.0 | 9 | 18.8 | 20 | 41.7 | 16 | 33.3 |  |
| **Q9** |  |  |  |  |  |  |  |  |  |  |  |  |  |  | 0.156 |
| **PSAN** | 0 | 0.0 | 0 | 0.0 | 2 | 2.5 | 4 | 5.1 | 7 | 8.9 | 16 | 20.3 | 50 | 63.3 |  |
| **AN** | 3 | 3.5 | 2 | 2.3 | 2 | 2.3 | 5 | 5.8 | 6 | 7.0 | 16 | 18.6 | 52 | 60.5 |  |
| **ND** | 0 | 0.0 | 1 | 2.1 | 0 | 0.0 | 1 | 2.1 | 9 | 18.8 | 15 | 31.3 | 22 | 45.8 |  |
| **Q10** |  |  |  |  |  |  |  |  |  |  |  |  |  |  | 0.593 |
| **PSAN** | 0 | 0.0 | 0 | 0.0 | 1 | 1.3 | 4 | 5.1 | 10 | 12.7 | 25 | 31.6 | 39 | 49.4 |  |
| **AN** | 2 | 2.3 | 2 | 2.3 | 2 | 2.3 | 6 | 7.0 | 9 | 10.5 | 30 | 34.9 | 35 | 40.7 |  |
| **ND** | 0 | 0.0 | 1 | 2.1 | 0 | 0.0 | 1 | 2.1 | 9 | 18.8 | 18 | 37.5 | 19 | 39.6 |  |
| **Q11** |  |  |  |  |  |  |  |  |  |  |  |  |  |  | 0.058 |
| **PSAN** | 2 | 2.5 | 2 | 2.5 | 4 | 5.1 | 7 | 8.9 | 20 | 15.3 | 25 | 31.6 | 19 | 24.1 |  |
| **AN** | 8 | 9.3 | 4 | 4.7 | 8 | 9.3 | 22 | 25.6 | 16 | 18.6 | 16 | 18.6 | 12 | 14.0 |  |
| **ND** | 3 | 6.3 | 1 | 2.1 | 5 | 10.4 | 8 | 16.7 | 13 | 27.1 | 7 | 14.6 | 11 | 22.9 |  |
| **Q12** |  |  |  |  |  |  |  |  |  |  |  |  |  |  | 0.079 |
| **PSAN** | 2 | 2.5 | 1 | 1.3 | 4 | 5.1 | 9 | 11.4 | 16 | 20.3 | 26 | 32.9 | 21 | 26.6 |  |
| **AN** | 6 | 7.0 | 5 | 5.8 | 12 | 14.0 | 18 | 20.9 | 14 | 16.3 | 18 | 20.9 | 13 | 15.1 |  |
| **ND** | 3 | 6.3 | 0 | 0.0 | 3 | 6.3 | 11 | 22.9 | 7 | 14.6 | 12 | 25.0 | 12 | 25.0 |  |
| **Q13** |  |  |  |  |  |  |  |  |  |  |  |  |  |  | **0.028** |
| **PSAN** | 1 | 1.3 | 1 | 1.3 | 5 | 6.3 | 9 | 11.4 | 15 | 19.0 | 19 | 24.1 | 29 | 36.7 |  |
| **AN** | 5 | 5.8 | 8 | 9.3 | 7 | 8.1 | 17 | 19.8 | 21 | 24.4 | 15 | 17.4 | 13 | 15.1 |  |
| **ND** | 3 | 6.3 | 0 | 0.0 | 4 | 8.3 | 8 | 16.7 | 11 | 22.9 | 11 | 22.9 | 11 | 22.9 |  |
| **Q14** |  |  |  |  |  |  |  |  |  |  |  |  |  |  | 0.159 |
| **PSAN** | 1 | 1.3 | 2 | 2.5 | 5 | 6.3 | 13 | 16.5 | 15 | 19.0 | 12 | 15.2 | 31 | 39.2 |  |
| **AN** | 9 | 10.5 | 5 | 5.8 | 6 | 7.0 | 15 | 17.4 | 14 | 16.3 | 20 | 23.3 | 17 | 19.8 |  |
| **ND** | 2 | 4.2 | 1 | 2.1 | 3 | 6.3 | 10 | 20.8 | 10 | 20.8 | 11 | 22.9 | 11 | 22.9 |  |
| **Q15** |  |  |  |  |  |  |  |  |  |  |  |  |  |  | 0.103 |
| **PSAN** | 0 | 0.0 | 2 | 2.5 | 4 | 5.1 | 14 | 17.7 | 11 | 13.9 | 18 | 22.8 | 30 | 38.0 |  |
| **AN** | 5 | 5.8 | 7 | 8.1 | 6 | 7.0 | 12 | 14.0 | 17 | 19.8 | 20 | 23.3 | 19 | 22.1 |  |
| **ND** | 2 | 4.2 | 1 | 2.1 | 1 | 2.1 | 9 | 18.8 | 13 | 27.1 | 13 | 27.1 | 9 | 18.8 |  |

PSAN: Psychosocial Sciences Applied to Nursing; AN: Ageing Nursing; ND: Nutrition and Dietetics.
